# Mutation and association analysis of the *PVR* and *PVRL2* genes in patients with non-syndromic cleft lip and palate

**DOI:** 10.1590/S1415-47572009000300007

**Published:** 2009-09-01

**Authors:** Mehmet A. Sözen, Jacqueline T. Hecht, Richard A. Spritz

**Affiliations:** Department of Medical Biology, School of Medicine, Afyon Kocatepe University, AfyonkarahisarTurkey; 2 Department of Pediatrics, University of Texas Medical School, Houston, TexasUSA; 3 Human Medical Genetics Program, University of Colorado Denver, Anschutz Medical Campus, Aurora, COUSA

**Keywords:** *PVR*, *PVRL2*, cleft lip and palate, mutation, SSCP

## Abstract

Orofacial clefts (OFC; MIM 119530) are among the most common major birth defects. Here, we carried out mutation screening of the *PVR* and *PVRL2* genes, which are both located at an OFC linkage region at 19q13 (*OFC3*) and are closely related to *PVRL1*, which has been associated with both syndromic and non-syndromic cleft lip and palate (nsCLP). We screened a total of 73 nsCLP patients and 105 non-cleft controls from the USA for variants in *PVR* and *PVRL2*, including all exons and encompassing all isoforms. We identified four variants in *PVR* and five in *PVRL2*. One non-synonymous *PVR* variant, A67T, was more frequent among nsCLP patients than among normal controls, but this difference did not achieve statistical significance.

Orofacial clefts (OFC) are common birth defects, occurring in approximately 1 per 800 North American Caucasian infants and varying in incidence among patients of different geographical origins (Vanderas, 1987). Most cases of OFC (70%) occur sporadically, and may include isolated cleft lip, cleft lip with or without palate (CLP) and isolated cleft palate. Such ‘non-syndromic' OFC appear to be polygenic in origin, a number of loci each exerting a relatively modest effect against a multi-factorial background (Spritz, 2001; Murray, 2002; Cobourne, 2004).

A role for one or more genes at chromosome 19q involved in non-syndromic CLP (nsCLP) has been supported by multiple lines of evidence. Genetic linkage studies defined *OFC3*, a region of genetic linkage to nsCLP on chromosome 19q13 (Stein *et al.*, 1995; Warrington *et al.*, 2006). Initial studies of candidate genes in the *OFC3* region focused on *BCL3*, a number of genetic association studies finding conflicting evidence of association with nsCLP in various populations (Stein *et al.*, 1995; Amos *et al.*, 1996; Maestri *et al.*, 1997; Gaspar *et al.*, 2002; Blanco *et al.*, 2004). Other genes studied in the *OFC3* region include *APOC2* (Marazita *et al.*, 2002), *CLPTM1* (Yoshiura *et al.*,1998; Turhani *et al.*, 2005; Warrington *et al.*, 2006), and *TOMM40* (Warrington *et al.*, 2006), each yielding, at best, inconsistent evidence for involvement in nsCLP.

Recent attention has turned to two other candidate genes in 19q13, *PVR* and *PVRL2*, which are paralogous to *PVRL1*, in which homozygous mutations result in a rare autosomal recessive CLP syndrome, CLPED1 (Suzuki *et al.*, 2000), and in which variants have been genetically associated with nsCLP in northern Venezuela (Sözen *et al.*, 2001) and perhaps other populations (Avila *et al.*, 2006; Scapoli *et al.*, 2006; Tongkobpetch *et al.*, 2008). *PVR*, *PVRL1*, and *PRVL2* respectively encode Necl-5/CD155, nectin-1 and nectin-2, cell adhesion molecules present at adherens junctions and other ectoplasmic specializations at cell-cell contacts (Young *et al.*, 2009), and are widely expressed during development. Warrington *et al.* (2006) reported genetic association of a rare intronic variant in *PVR*, C__1828143_10 (rs35385129), with nsCLP in patients from several different populations, and by direct sequencing identified 7 variants in *PVR* and 16 in *PVRL2*, none of which appeared to be causal for nsCLP. A subsequent study found marginal association of nsCLP in Italian patients with *PVR* SNP rs35385129, but no association with a single intronic *PVRL2* SNP analyzed (Pezzetti *et al.*, 2007).

In the present study, we carried out mutation analyses of *PVR* and *PVRL2* in USA Caucasian (CEU) patients with nsCLP and non-cleft controls. We obtained genomic DNA samples with informed consent from USA patients with nsCLP but with no other physical or cognitive abnormalities, and from unaffected controls. We initially screened all 8 exons of *PVR* and 10 exons of *PVRL2* (encompassing all known mRNA isoforms, except the last 21 nt of *PVRL2* exon 1) in 73 unrelated USA CEU nsCLP patients and 105 unrelated USA CEU controls. Subsequently, an additional 28 unrelated USA CEU nsCLP patients were specifically analyzed for the *PVR* rs1058402 (A67T) variant. The *PVR* and *PVRL2* PCR primers used are given in Table 1. PCR products were screened for variation by simultaneous single-stranded conformation polymorphism (SSCP)/heteroduplex analysis by electrophoresis in 0.5X MDE gels (Biowhittaker Molecular Applications) containing 10% glycerol (Lee *et al.*, 1995), followed by DNA sequence analysis of those amplicons exhibiting aberrant SSCP/heteroduplex patterns.

As shown in Table 2, we identified a total of four variants in *PVR*: rs11540085 (-1C > T), rs1058402 (A67T), rs203710 (I340M), and one novel variant, 19:49856876G > A (E404E). Similarly, we identified a total of five variants in *PVRL2*: rs41290128 (D496N), rs283814 (P409P) and three novel variants, 19:50077328G > A (A355T), 19:50081289T > C (F440F) and 19:50073660_50073661insAGG (R461-462ins). Most variants were observed in both patients and controls, except for the rare variant *PVR* 19:49856876G > A (E404E), which was observed in only one control, *PVRL2* 19:50077328G > A (A355T) and 19:50081289T > C (F440F), which we only observed in one and two patients, respectively, and 19:50073660_50073661insAGG (R461-462ins), which was observed in two controls. The allele frequency of the non-synonymous *PVR* variant, rs1058402 (A67T), in spite of being somewhat greater among nsCLP patients (0.039) than among controls (0.014), did not achieve statistical significance (p = 0.098). Likewise, genotype frequency distribution of the rs1058402 variant was not significantly different between nsCLP patients and controls.

The etiology of orofacial clefts is complex, most likely involving many different genetic and environmental factors, most of which remain unknown. Genetic association and linkage, besides studies, have implicated a region in chromosome 19q13, termed *OFC3*. Warrington *et al.* (2006) reported genetic association between nsCLP and a rare intronic variant in *PVR*, rs35385129, in two distinct populations, although no association was found in two other patient groups (Warrington *et al.*, 2006; Pezzetti *et al.*, 2007). In spite of the statistically non-significant results here reported, our data suggest that *PVR* may bear further investigation.

## Figures and Tables

**Table 1:** Oligonucleotide primers for PCR amplification of the exons of *PVR* and *PVRL2*.

Amplicon	Primer sequences	Amplicon size (bp)
*PVR*		
Exon 1	5' AGAGCGACGGGCGCCGGGAA 3' 5' ACTGCGCGGGGGTCACTCAC 3'	165
Exon 2	5' TTCTCTTCGGTTCTCCGCAG 3' 5' CCCCAAAACCCCCTGCTC 3'	388
Exon 3	5' GCTTTTGTTCCTCTTCCCAG 3' 5' GCTGACTTGGGCACACTCAC 3'	337
Exon 4	5' TCTGTATCCATTTCCTGCAG 3' 5' CCCTGAGACCCAGGACTCAC 3'	158
Exon 5	5' CACCTTTCTGTCTCTCTCCCAG 3' 5' CCACCCAGGGAGTTCCTCAC 3'	189
Exon 6	5' CCTGTTTCCTTCTCTTTCAG 3' 5' GTAGGTGCTCAATTACGGCA 3'	216
Exon 7	5' TTCCCCTCCTATTTCCCCAG 3' 5' AGCTCCAACACTGCACTTAC 3'	72
Exon 8	5' ATTTGAAAACCCTCTTCTAG 3' 5' GGTCCAACTCTGGAGGCCCA 3'	158

*PVRL2*		
Exon 1	5' CTACTAAACCGCCCAGCCGA 3' 5' CGGTTTCCAGGAGCAGCAGC 3'	169
Exon 2	5' GTGGCCCTGCCTGGAGGTGT 3' 5' TGACCCGCAAGGGGATGCTC 3'	530
Exon 3	5' CTCCTCTGCTGAGTGTTTGT 3' 5' GTAGACAGTGCTTTAGAGAA 3'	437
Exon 4	5' CTATCTGCTAACTTGTCCAC 3' 5' TTAGATCCAGGAGTCCAGGC 3'	258
Exon 5	5' TCTTTAGGGATGAGGCCTGTG 3' 5' AAGTCCTGAAGGGCAGAACT 3'	290
Exon 6	5' CCCAGAGCGATCCTCGTGAT 3' 5' AACCAGTCTGGAACCCTAGG 3'	550
Exon 7	5' GATGGTCGCTTGGAATAAGG 3' 5' CTCACCCTACCCCATACTC 3'	295
Exon 8	5' GTGCCATAACCCCGGAGTCA 3' 5' GCCAGGCCCCTCCCAGCCCT 3'	205
Exon 9	5' GGCCTGGCAGGGAGAAGCTG 3' 5' TTGCCAGGCTTGACCCCTGG 3'	228
Exon 10	5' AAGAGCAGATTGGTAATCTG 3' 5' GGCACTAGATCCTTGGCAAG 3'	411

**Table 2:** *PVR* and *PVRL2* variants observed in USA CEU nsCLP patients and controls.

Variants*	Allele frequency				Genotype frequency***							
	Cases	Controls	p-value**		Cases				Controls			p-value***
					11	12	22		11	12	22	
*PVR*												
rs11540085 (-1C > T)	1/146 (0.007)	1/210 (0.005)	0.653		72	1	0		102	3	0	0.645
rs1058402 (A67T)	8/202 (0.039)	3/210 (0.014)	0.098		94	6	1		102	3	0	0.245
rs203710 (I340M)	4/146 (0.027)	3/206 (0.015)	0.317		69	4	0		100	3	0	0.451
19:49856876G > A (E404E)	0/146 (0)	1/178 (0.006)	0.549		73	0	0		88	1	0	1.000

*PVRL2*												
19:50077328G > A (A355T)	1/144 (0.007)	0/144 (0)	0.500		71	1	0		72	0	0	1.000
rs283814 (P409P)	3/144 (0.068)	1/144 (0.007)	0.311		69	3	0		71	1	0	0.620
19:50081289T > C (F440F)	2/144 (0.027)	0/202 (0)	0.173		70	2	0		101	0	0	0.172
19:50073660_50073661insAGG (R461-462ins)	0/146 (0)	2/206 (0.001)	0.342		73	0	0		101	2	0	0.512
rs41290128 (D496N)	2/144 (0.027)	2/202 (0.001)	0.553		70	2	0		99	2	0	1.000

*Nucleotide positions are referent to NCBI Build 36 (November, 2005), release 38 (April, 2006). **2X2 Fisher's exact test, 1-tailed assuming that the minor allele tags a potential risk variant. ***For each SNP, the major allele was designated 1 and the minor allele was designated 2; 2X3 Freeman-Halston extension of Fisher's exact test, 2-tailed. p-values are given without Bonferroni correction.
